# Novel Therapeutic Approaches in Inherited Neuropathies: A Systematic Review

**DOI:** 10.3390/pharmaceutics15061626

**Published:** 2023-05-30

**Authors:** Manon Hustinx, Ann-Marie Shorrocks, Laurent Servais

**Affiliations:** 1 Department of Paediatrics, MDUK Oxford Neuromuscular Centre and, NIHR Oxford Biomedical Research Centre, University of Oxford, Oxford OX1 3DW, UK; ann-marie.shorrocks@worc.ox.ac.uk (A.-M.S.); laurent.servais@paediatrics.ox.ac.uk (L.S.); 2 Centre de Référence des Maladies Neuromusculaires, Department of Neurology, University Hospital Liège, and University of Liège, 4000 Liège, Belgium; 3 Centre de Référence des Maladies Neuromusculaires, Department of Paediatrics, University Hospital Liège, and University of Liège, 4000 Liège, Belgium

**Keywords:** inherited neuropathies, treatment, disease-modifying therapy

## Abstract

The management of inherited neuropathies relies mostly on the treatment of symptoms. In recent years, a better understanding of the pathogenic mechanisms that underlie neuropathies has allowed for the development of disease-modifying therapies. Here, we systematically review the therapies that have emerged in this field over the last five years. An updated list of diseases with peripheral neuropathy as a clinical feature was created based on panels of genes used clinically to diagnose inherited neuropathy. This list was extended by an analysis of published data by the authors and verified by two experts. A comprehensive search for studies of human patients suffering from one of the diseases in our list yielded 28 studies that assessed neuropathy as a primary or secondary outcome. Although the use of various scales and scoring systems made comparisons difficult, this analysis identified diseases associated with neuropathy for which approved therapies exist. An important finding is that the symptoms and/or biomarkers of neuropathies were assessed only in a minority of cases. Therefore, further investigation of treatment efficacy on neuropathies in future trials must employ objective, consistent methods such as wearable technologies, motor unit indexes, MRI or sonography imaging, or the use of blood biomarkers associated with consistent nerve conduction studies.

## 1. Introduction

Hereditary neuropathies are a broad range of diseases that differ in terms of inheritance (dominant, recessive, X-linked, or mitochondrial), electrophysiological features (demyelinating, axonal, or intermediate), and clinical properties. Indeed, neuropathy may be the primary feature of a disease, such as in Charcot–Marie–Tooth disease (CMT), hereditary sensory and autonomic neuropathies (HSAN), or hereditary motor neuropathies (HMN), in which patients may present different levels of muscle atrophy and weakness, sensory and autonomic disturbances, and/or skeletal deformities. On the other hand, neuropathy may be part of a more complex systemic disease, as in peroxisomal, lysosomal, or mitochondrial diseases, for which neuropathy can be the first symptom but can also remain subclinical, making diagnosis more challenging [1,2,3]. The global prevalence of inherited neuropathies is unknown, but CMT has an estimated prevalence of 1/2500, making it the most common inherited disorder of the peripheral nervous system. The management of inherited neuropathies relies mostly on symptomatic treatments such as physiotherapy, analgesics, or surgery [4]. In recent years, a better understanding of the pathological mechanisms underlying some of these diseases has allowed the development of disease-modifying therapies or diets, although these treatments have been validated for a very small subset of diseases [2,5,6]. Well-conducted reviews have been published regarding the management of inherited neuropathies [7,8]; however, the ever-growing landscape of these diseases means that these reviews are outdated. We conducted a review of the recent literature to identify approaches for the treatment of primary hereditary neuropathies and also more complex systemic diseases with peripheral neuropathy as a clinical feature. Our systematic review demonstrates that patients with certain inherited neuropathies benefit from specific treatment and that objective, consistent methods for the evaluation of responses to treatment are needed.

## 2. Methods

### 2.1. Compilation of List of Inherited Neuropathies

Two authors (MH and A-MS) identified genes used clinically to determine inherited neuropathies by merging genes from panels used in the UK (PanelApp) [9], France (Paris-Saclay) [10], and the USA (Mayo Clinic) [11]. These genes were merged into one list and linked to specific diseases using Orphanet [12] and Genecards [13] databases. The list was then extended based on the expertise of the authors and two experts (Dr Tania Stojkovic, Nord/Est/Ile-de-France Neuromuscular Reference Center, Institut of Myology, Pitié-Salpêtrière Hospital, APHP, Sorbonne University, Paris, France, and Dr Isabelle Lievens, Neuromuscular Reference Center, Department of Neurology, University Hospital Liège and University of Liège, Liège, Belgium). Finally, our results were crossed with existing published data from Fernandez-Eulate et al. [2], Rossor et al. [1], Masingue et al. [3], and Finsterer et al. [14]. Appendix A provides the list of genes associated with inherited neuropathies.

### 2.2. Literature and Clinical Trial Database Searches

**Figure 1:**
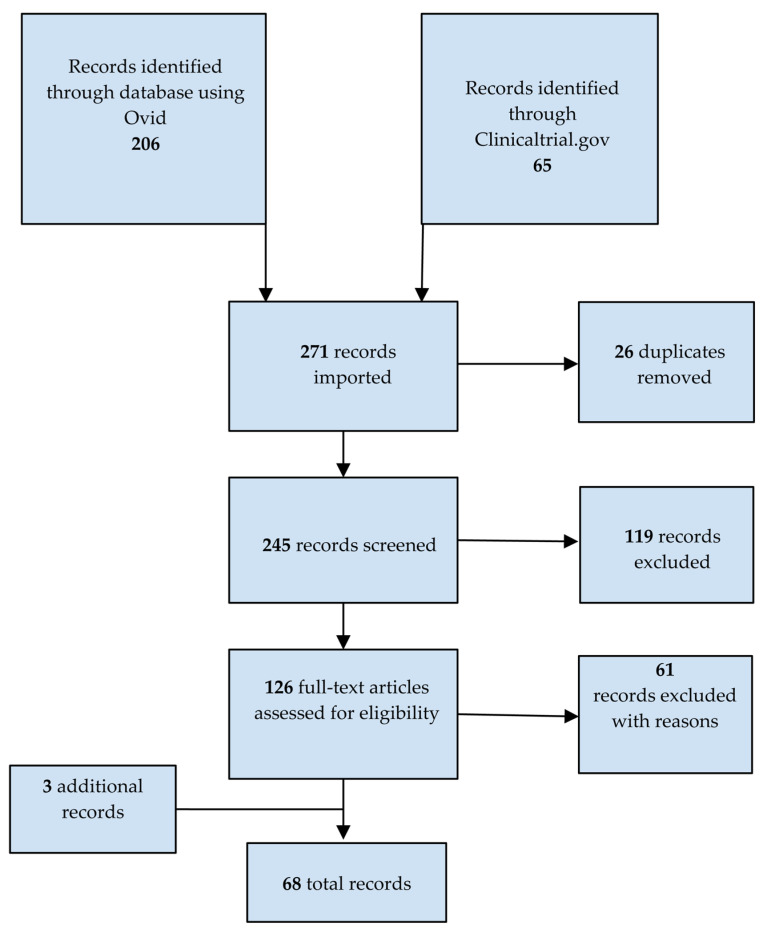
PRISMA flowchart of studies identified as relevant to the treatment of inherited neuropathies.

The Preferred Reporting Items for Systematic Reviews and Meta-Analyses (PRISMA) checklist and flow diagram were followed for the design and reporting of this work, as detailed below. Two independent authors (MH and A-MS) conducted a systematic search of Ovid Medline [15] on 24 February 2023 for original, full-text articles published in English after 1 January 2018. The studies that were included described the assessment of treatments in any of the inherited neuropathies identified (Appendix A) with a clinical, biological, or electrophysiological outcome. Non-pharmacological interventions such as physiotherapy, genetic counselling, or surgery were excluded. Retrospective observational studies were not included. A search was also carried out using the Clinicaltrial.gov [16] database on 24 February 2023. Database-specific filters were applied to ensure that only interventional studies completed with results published after 1 January 2018 were included and that studies describing diseases such as cancer, carpal tunnel, amyloid cardiomyopathy, X-linked cerebral adrenoleukodystrophy, and bone assessment in Gaucher disease were not included. Finally, relevant articles were extracted from the reference sections of systematic or expert reviews and meta-analyses. The search strategy is shown schematically in Figure 1. A table displaying information about the diseases for which clinical trials were identified is presented in Appendix B.

### 2.3. Selection of Studies

Two researchers (MH and A-MS) independently screened titles and abstracts and then full texts for eligibility. To be included, studies had to meet the following criteria: (1) the study was performed on human patients, (2) patients had been diagnosed with one of the diseases included in our list (Appendix A), (3) a pharmacological treatment or diet was evaluated, and (4) the results were presented. No restriction was applied regarding gender, age at onset, severity, ethnicity, or phenotypic features. The two reviewers compared their findings and potential disagreements were resolved by consensus.

### 2.4. Data Extraction and Analysis

For each study that fulfilled our criteria, information was collected on the study (authors, year of publication, type, duration), the population (sample, specific characteristics where applicable), the treatment (drug/dietary supplement name, dosing and administration, mechanism), and the outcome (clinical, biological, or electrophysiological). Clinical and biological outcomes that were related to symptoms other than neuropathies were not extracted. All biomarkers were included as they were considered a way of attesting to the disease-modifying aspect of treatments. Only the most recent updates on pilot projects were considered. Eligible papers were assessed for risk of bias using the Jadad score (score ranging from 0 to 5 according to whether the study was described as randomized and double-blinded and including a thorough description of withdrawals) [17] (Appendix C). Data extraction was carried out by MH, reviewed by A-MS, and then reviewed by LS. Due to differences in clinical and biological outcomes, meta-analysis was precluded.

## 3. Results

### 3.1. Identification of Studies That Assessed Treatments for Inherited Neuropathies

We first compiled a list of genes associated with neuropathies. Based on a literature and clinical trials database search for studies of pharmacological treatments or dietary interventions in patients diagnosed with one of the diseases identified in our list (Appendix A), we identified 271 studies, from which 26 duplicates were removed. Of the remaining studies, 119 were excluded based on titles and abstracts. The remaining 126 studies were read in full by two assessors, resulting in the exclusion of 61 studies. Three additional relevant studies were captured from the reference sections of systematic or expert reviews and meta-analyses. In the end, 68 studies met our criteria (Figure 1). Among these, only 28 assessed neuropathy as a primary or secondary outcome. These studies assessed 14 different drugs in nine inherited neuropathies (Table 1).

Moreover, 40 articles that assessed biomarkers or neurological clinical aspects potentially related to neuropathy were identified (Table 2). Changes in disease-related biomarkers or the modification of neurological features such as balance, gait, strength, or dexterity can be indicative of therapeutic efficacy, even if neuropathy was not assessed as a primary or secondary outcome. The inclusion of biomarkers or neurological clinical aspects potentially related to neuropathy allowed us to capture early-phase, proof-of-concept studies.

Appendix B lists modes of inheritance, clinical presentation, and standards of care for each disease for which a relevant clinical trial was identified.

### 3.2. Treatments for Conditions Where Neuropathy Is the Sole or Predominant Feature of the Disease

#### 3.2.1. Familial Dysautonomia

A randomized, placebo-controlled, crossover phase 2 study of the effect of DOPA decarboxylase inhibitor carbidopa was conducted in 22 patients with familial dysautonomia [41]. At the end of the three 4-week treatment periods, the two co-primary endpoints, reduction in systolic blood pressure variability and systolic blood pressure peaks, were met. Although this is a symptomatic treatment, this could improve quality of life.

#### 3.2.2. Hereditary Sensory and Autonomic Neuropathy Type 1 (HSAN1)

A randomized, placebo-controlled, phase 1/2 study of L-serine with an open-label extension (OLE) was conducted on 18 patients with HSAN1 [42]. The total duration of the trial was 2 years. This study showed a non-significant improvement in the Charcot–Marie–Tooth Neuropathy Score (CMTNS) compared to placebo, a significant reduction in the neurotoxic deoxysphinganine levels, and evidence of reinnervation at 1 year from distal biopsies. No significant electrophysiological differences were detected in the tested nerves after 1 year; however, there was a paucity of recordable responses at baseline, and the statistical power of the study was limited by the small sample size. Further studies are needed to determine the efficacy of this potentially disease-modifying treatment.

#### 3.2.3. Charcot–Marie–Tooth 1A (CMT1A)

After a favourable phase 2 trial [94], a randomized, double-blind, placebo-controlled phase 3 study was conducted to evaluate the efficacy and safety of a high- and low-dose combination of baclofen, naltrexone, and sorbitol (PTX3003) in 323 CMT1A patients over 15 months. Although crystal formation in the high-dose formulation led to early discontinuation, significant clinical improvements were observed in this group compared to the placebo. In the ongoing open-label continuation study, participants are being treated with twice the volume of the low-dose formulation instead of the high dose to limit this stability issue [43].

### 3.3. Treatments for Conditions Where Neuropathy Is One Symptom of the Disease

#### 3.3.1. Hereditary Transthyretin Amyloidosis Polyneuropathy (ATTRv-PN)

**Transthyretin (TTR) stabilizers:** The safety and efficacy of tafamidis in delaying neurologic disease progression in patients with early-stage ATTRv-PN (stage 1) have been demonstrated in several clinical trials, extension studies [95], and around 10 years of clinical follow-up [18]. Tafamidis was approved in Europe in 2011 and subsequently in several other countries for the treatment of stage 1 ATTRv-PN. This drug has not been approved in the USA for this condition due to a lack of robust efficacy in clinical trials [21].

Tolcapone is another TTR stabilizer approved for clinical use in the treatment of Parkinson’s disease. It is currently under development to treat ATTRv-PN and other forms of ATTR, including leptomeningeal amyloidosis, due to its ability to cross the blood–brain barrier [56]. It was shown to have efficacy in a phase 2 study of subjects with ATTRv-PN [55].

Acoramidis (AG10) is an orally bioavailable, selective TTR stabilizer that is currently being assessed for effects on mortality and hospitalization for cardiovascular causes in subjects with transthyretin amyloid cardiomyopathy in an ongoing phase 3 trial, with topline data expected in mid-2023. The phase 3 study in ATTRv-PN patients was terminated by the sponsor after a review of the currently available treatments [53,96,97].

**Gene silencers:** Inotersen, which is an antisense oligonucleotide that targets TTR mRNA for degradation, proved its efficacy in a 15-month, randomized, placebo-controlled, double-blind phase 3 trial conducted on 172 adult patients with stage 1 or 2 ATTRv-PN [31,32,33,34]. Glomerulonephritis occurred in three patients and thrombocytopenia occurred in three patients treated with inotersen, with one death associated with grade 4 thrombocytopenia [31]. In the OLE, an update at 3 years showed a sustained efficacy compared with natural history, regardless of the mutation, prior treatment, or stage of the disease (except in the group treated first with the placebo and then inotersen in stage 2). In the OLE, no cases of acute glomerulonephritis were reported, but approximately 50% of patients in both the group treated first with the placebo and then with inotersen and the group continuously treated with inotersen experienced thrombocytopenia (<100 × 10^9^/L), although none had a grade 4 platelet count decrease [36]. Narayanan et al. [98] reported that patients with grade 4 thrombocytopenia in the phase 3 trial had higher levels of proinflammatory cytokines at baseline, which suggests a predisposition to immune-mediated thrombocytopenia via antiplatelet IgG antibodies. A post hoc analysis demonstrated that the higher health-related quality of life of patients treated with inotersen compared to placebo-treated subjects during the pivotal study was sustained through week 104 of the OLE study, highlighting the importance of early treatment [37].

Eplontersen is an antisense oligonucleotide targeting TTR mRNA that is conjugated to N-acetylgalactosamine, a ligand for a receptor expressed on hepatocytes, which improves distribution to these liver cells compared to unconjugated oligonucleotides. Two randomized, double-blind, placebo-controlled phase 1 studies showed greater TTR reduction compared to inotersen (88% at 45 mg q4w relative to inotersen 74% at 300 mg weekly) [51]. The efficacy and safety of eplontersen are currently being investigated in phase 3 trials.

Patisiran is a small interfering RNA (siRNA) that targets TTR mRNA for degradation through the endogenous RNA interference pathway to reduce the expression of TTR [22]. The efficacy and safety of patisiran were assessed in an 18-month randomized, placebo-controlled, double-blind phase 3 trial in 225 adult patients with a polyneuropathy disability score of IIIb or lower. Positive effects were seen across all subgroups, irrespective of age, sex, race, body weight, mutation, prior use of tafamidis, and mild/moderate renal or hepatic involvement [24]. Most of the adverse events were mild or moderate and the rate of serious adverse events was similar between the patisiran-treated and placebo groups [22]. Another 24-month phase 2 OLE study confirmed this favourable safety and efficacy profile, with concomitant tafamidis or diflunisal use [25]. A phase 3b open-label trial proved efficacy and tolerability in subjects with ATTRv-PN after liver transplantation, and no apparent drug interactions were observed between patisiran and immunosuppressive treatments [27].

Vutrisiran is another siRNA that reduces the synthesis of variant and wild-type transthyretin. Unlike patisiran, it is given subcutaneously and hence does not require premedication. In a phase 3 open-label study, vutrisiran reduced TTR levels to an extent similar to patisiran and was more efficacious than patisiran in multiple assessments. Vutrisiran was well tolerated and most adverse events were consistent with an expected ATTRv natural history progression [29]. A randomized treatment extension period is currently ongoing in which patients receive vutrisiran injections once every 6 months or once every 3 months.

**Gene editing:** NTLA-2001 is a gene-editing therapy based on CRISPR-Cas9. The two active components of this therapeutic are the mRNA encoding the Cas9 endonuclease and a single guide RNA complementary to the gene encoding TTR. These components are encapsulated in a lipid nanoparticle that enables targeted delivery to hepatocytes. The interim results of an ongoing phase 1 study (NCT04601051) in six patients with ATTRv-PN showed a reduction in TTR concentration in a dose-dependent manner after a single intravenous dose. In terms of safety, all the adverse events occurring during or after treatment were mild in severity. Increased D-dimer levels were noticed 4 to 24 h after infusion in five of the six patients but values returned to baseline after 7 days [54,97].

**Comparison of approved ATTRv-PN treatments:** No head-to-head comparisons of the therapeutics approved for the treatment of ATTRv-PN have been made. Indirect comparisons based on available data suggest that patisiran has a greater effect on neuropathy and quality of life than inotersen or tafamidis [99,100]. The choice of treatment should be based on the physician’s expertise, considering the disease’s stage and phenotype and country-specific recommendations. Further questions that need to be addressed include the optimal timing of treatment initiation or whether combinations of treatments might be more efficacious than single agents.

#### 3.3.2. Metachromatic Leukodystrophy (MLD)

Enzyme replacement therapy (ERT) with recombinant human arylsulfatase A (rhASA) has been tested in two phase 1/2 open-label trials in subjects with MLD, one with intravenous (IV) delivery and one with intrathecal (IT) delivery [44,45]. The study assessing the IV delivery of ERT was conducted in a cohort of 13 children. The trial lasted 52 weeks, and there was an additional 24-month extension period. The study with the IT delivery of ERT, on the other hand, enrolled 24 children and lasted for 38 weeks. The ongoing extension study should be completed by December 2024 (NCT01887938). Both ERT dosing routes resulted in a reduction in cerebrospinal fluid (CSF) sulfatide levels and no serious drug-related adverse events, but a clinical improvement was not demonstrated and electrophysiological parameters did not change compared to baseline. The IV study was terminated after 24 months due to a lack of efficacy, suggesting that the recombinant protein may not cross the blood–brain barrier in therapeutic quantities. In the study evaluating the IT delivery of ERT, 10 patients developed anti-rhASA antibodies in serum, with seven having an in vitro neutralizing status, but no correlation was found with the occurrence of adverse events. Anti-rhASA antibodies were also detected in CSF in some patients but a neutralizing status has not been determined. 

A gene therapy, atidarsagene autotemcel (arsa-cel), has also been tested in subjects with MLD. The therapeutic is an autologous haematopoietic stem and progenitor cell (HSPC) population transduced ex vivo with a lentiviral vector encoding ASA. This novel approach was evaluated in a phase 1/2 study conducted on 29 children with pre-symptomatic or early-symptomatic early-onset MLD compared to a natural history cohort [46]. Promising results were obtained, such as increased ARSA activity and considerable clinical improvement. Some patients were found to have gross motor development similar to healthy children. There were significant differences in nerve conduction velocities between treated patients with late-infantile MLD and the natural history cohort. In terms of safety, arsa-cel was well tolerated with busulfan conditioning. Four patients developed anti-ARSA antibodies but this did not affect clinical outcomes.

#### 3.3.3. Spinal and Bulbar Muscular Atrophy (SBMA or Kennedy Disease)

Patients with SBMA may experience myotonia-like symptoms under cold exposure. It has been hypothesized that this is linked to sodium channel dysfunction in skeletal muscles [101]. Yamada et al. [49] conducted an observational study on 51 SBMA patients and noticed that ulnar nerve distal latency was prolonged under cold exposure compared to healthy controls (*p* < 0.001), and this was correlated with grip strength. They then conducted a randomized, placebo-controlled, double-blind study on 20 subjects over 4 weeks to evaluate mexiletine hydrochloride. Statistically significant improvements in some clinical parameters were observed, although there was not a significant change in nerve conduction velocity. No serious adverse events were reported. Further trials are warranted to evaluate the symptomatic effect of mexiletine hydrochloride on motor function.

Insulin-like growth factor 1 (IGF-1) stimulates Akt-mediated phosphorylation of the androgen receptor, which promotes its clearance. BVS857 is a pegylated IGF-1 mimetic with a longer half-life than endogenous IGF-1. The safety and efficacy of BVS857 were evaluated in a randomized, placebo-controlled trial in 27 adults that lasted 12 weeks. The subcutaneous delivery of BVS857 resulted in erythema at the injection site and low and variable exposure. Thus, IV delivery was employed in phase B of the study. Five participants developed neutralizing antibodies to endogenous IGF-1, but this did not have a clinical or pharmacokinetic impact. The half-life of BVS857 was less than theorized, barely 24 h. Nevertheless, a significant increase was observed in thigh muscle volume over the short period of the study, although this was not correlated with the improvement of any of the motor functional measures [90].

#### 3.3.4. Adult Polyglucosan Body Disease (APBD)

Polyglucosan bodies were detected in the astrocytes of a patient with confirmed glycogen branching enzyme (GBE) deficiency [102]. Therefore, it is believed that the accumulation of abnormal glycogen in astrocytes leads to a lack of energy substrates for neurons and is responsible for certain symptoms of APBD. Due to its anaplerotic properties, triheptanoin was tested in a randomized, cross-over, placebo-controlled trial on 23 participants with APBD over 1 year (6 months on treatment and 6 months on placebo), followed by a 4-year OLE. Neither the 6 min walk test (6MWT) nor the secondary endpoints were met at the end of the study. However, two patients who were mildly disabled at baseline remained clinically stable after treatment discontinuation, as assessed by the 6MWT and Expanded Disability Status Scale (EDSS), and this was associated with stable nerve conduction studies under treatment. This study confirmed the safety profile of triheptanoin, but further investigations are needed to evaluate its effect on peripheral nerve deficits [50].

#### 3.3.5. Fabry Disease (FB)

The mechanisms underlying the neuropathy experienced by FB patients are not fully understood, but globotriaosylceramide (Gb-3) has been demonstrated to accumulate in dorsal root ganglia [103,104], myelinated axons [105], and microvascular endothelial cells, which can result in ischemic axonal degeneration [106]. These data suggest that Gb3 drives peripheral neuron dysfunction. Two approved ERTs (agalsidase alfa and beta) have shown favourable effects on peripheral neuropathies and reduce Gb3 and globotriaosylsphingosine (lyso-Gb3), especially when initiated soon after the onset of symptoms [107,108,109]. Lower dose regimens of agalsidase beta were tested in a paediatric cohort by Ramaswami et al., but it did not show consistent benefit, and the approved dose of 1 mg/kg/2-weekly was supported by this trial [62]. More recently, a new agalsidase beta (ISU303) with an almost identical structure to Fabrazyme was developed by ISU Abxis. This alternative agalsidase beta is of lower cost, but close monitoring is required to ensure its quality, safety, and efficacy [63]. A PEGylated ERT, pegunigalsidase alfa, was developed with the aim of increasing plasma half-life and reducing immunogenicity. A 1-year, dose-ranging, open-label, phase 1/2 trial (NCT01981720) of the PEGylated compound was conducted on 18 adult FB patients [64]. One patient was withdrawn from the study due to bronchospasm after the first infusion, and three patients developed treatment-induced antibodies, but this did not influence the efficacy or safety. The results of the 60-month extension study were recently published on clinicaltrial.gov, but no peer-review publications were available at the time of this review.

Migalastat is an approved orally administered chaperone that enhances the activity of endogenous patients’ alpha-galactosidase-A. It is only suitable for selected patients with certain mutations, as determined by an in vitro assay [110]. Results from the ATTRACT trial, an 18-month randomized, open-label, active-controlled trial and its 12-month OLE showed a relatively safe profile among adult FB patients after a switch from an ERT to migalastat. Migalastat had comparable or superior effects on renal function, reducing cardiac mass and FB-related clinical events, compared to the ERT. The effect on neuropathy was not monitored in this trial. Interestingly, plasma lyso-Gb3 levels remained low from baseline to month 30 in migalastat-treated subjects. White blood cell alpha-galactosidase-A activity in male patients increased in the group treated with migalastat during the randomized period but remained stable in the group treated with the ERT [58,59]. The efficacy of migalastat in males with classic phenotypes was confirmed in the phase 3 FACETS trial and its extension study, but, again, the impact on neuropathy was not assessed [60].

Venglustat, a substrate reduction therapy, was assessed in a 26-week open-label phase 2 study and its 130-week extension study in 11 ERT-naïve adult male patients with classic FB phenotypes. Biomarkers all decreased upon treatment with venglustat, but the effect on pain was not significantly sustained at the end of the study and specific neuropathy endpoints were not assessed [61]. Phase 3 trials are ongoing.

#### 3.3.6. Acute Intermittent Porphyria (AIP)

A siRNA that silences the expression of the gene encoding ALAS1 has been approved for the treatment of AIP [111]. The siRNA, givosiran, prevents the accumulation of delta-aminolevulinic (ALA) and porphobilinogen (PBG), which are intermediates in heme synthesis. In the ENVISION trial, givosiran treatment resulted in sustained reductions in hepatic ALAS1 mRNA and urinary ALA and PBG levels and annualized attack rate compared to placebo [74,76]. Givosiran had an acceptable safety profile, although two patients discontinued the study because of increased homocysteine levels and one because of alanine aminotransferase levels greater than eight times the upper limit of normal. Small decreases in eGFR observed early in therapy stabilized over months 12 to 24, and no patients discontinued givosiran due to renal events during the extension period [75]. By lowering the annualized attack rate, givosiran reduces the probability that patients will develop acute motor neuropathy during an attack or more chronic neuropathies that appear in subjects not treated promptly or with recurrent attacks [112].

### 3.4. Treatments for Conditions Where Neuropathy Plays a Role in Ataxia

#### 3.4.1. Spinocerebellar Ataxia Type 38 (SCA 38)

It has been shown that mutations in *ELOVL5* are responsible for a reduction in serum docosahexanoic acid (DHA) in patients with SCA 38 [113]. Oral DHA given to nine patients in a 2-year OLE study showed clear clinical benefits and nerve conduction velocities were stable during the treatment period [40]. To determine whether DHA has an effect on neuropathy, additional information, such as analyses of compound muscle action potential and sensory nerve action potential, which are relevant considering the predominantly axonal nature of the polyneuropathy experienced by SCA 38 patients [114], and a longer follow-up are needed.

#### 3.4.2. Spinocerebellar Ataxia Type 2 (SCA2)

Two randomized, placebo-controlled, double-blind studies were recently conducted in SCA2 cohorts. The first assessed the safety and efficacy of riluzole, which had previously shown benefits in inherited ataxias [115]. The study was conducted over one year in 45 moderately affected adult patients; riluzole treatment did not result in any improvement compared to placebo [65]. The second study enrolled 34 mildly or moderately affected Cuban adult patients to test the efficacy and safety of nasally administered human recombinant erythropoietin (EPO). Supporting the utility of this treatment, endogenous EPO is abnormally low in the cerebrospinal fluid (CSF) of SCA2 patients [116], and EPO has been shown to have an additional neurotrophic role [117]. The proportion of high responders in the spinocerebellar ataxia functional index (SCAFI ≥ 0.75), which is a composite score of motor performances, was slightly higher among the EPO-treated patients than those given a placebo despite a considerable placebo effect. Moreover, the drug was relatively well tolerated including in terms of erythropoiesis activity [66]. Further studies are warranted to evaluate the effect of EPO on neuropathy.

#### 3.4.3. Ataxia-Telangiectasia (A-T)

The efficacy of low-dose betamethasone was studied in a 2-year open-label study of patients with A-T. Although transient efficacy was observed after 6 months, sustained benefits were not observed at the end of the trial [87]. Although it was not used in this specific trial, the encapsulation of steroids within autologous erythrocytes (EryDex) allows a slow release for up to 1 month. This novel encapsulation approach was used with dexamethasone sodium phosphate in a phase 3, randomized-controlled, double-blind trial (ATTeST) in A-T patients and showed clear clinical benefits after 6 months [88].

#### 3.4.4. X-Linked Adrenoleukodystrophy (X-ALD)

Redox imbalance plays an important role in the pathogenesis of X-ALD, and this is the reason why a combination of antioxidants (α-tocopherol, N-acetylcysteine, and α-lipoic acid) was evaluated in a pilot open-label phase 2 study in 13 subjects [48]. In patients with X-ALD, there is a negative correlation between the levels of the chemokine MCP1 and the pro-inflammatory metabolite 15-hydroxyeicosatetraenoic with 6MWT, hence these oxidative damage markers are considered predictors of disease progression. Treatment with the combination of antioxidants resulted in a considerable decrease in all oxidative damage markers and pro-inflammatory markers and significant clinical improvements. Nerve conduction velocities were stable after 1 year in treated patients, whereas the conduction time of motor-evoked potential decreased, suggesting an improvement in upper motor neuron function. Laser-evoked potentials increased in two-thirds of treated patients. Although these positive effects do not seem to be linked to an impact on neuropathy, further testing of antioxidants is warranted.

ADVANCE was a randomized, double-blind, phase 2/3 study evaluating the safety and efficacy of oral leriglitazone in 116 men with X-ALD who had adrenomyeloneuropathy for a total of 96 weeks [89]. A change from baseline in the 6MWT, the primary outcome measure, was not observed. Therefore, the hierarchical testing of all secondary endpoints was not performed. Moreover, adverse effects, including weight gain and peripheral oedema, were observed. In post hoc subgroup analyses, patients with early-stage disease who were treated with leriglitazone had less decline in the 6MWT and in EDSS scores relative to those treated with a placebo at week 96. Finally, no leriglitazone-treated patients developed cerebral adrenoleukodystrophy (CALD), demonstrating that the drug may slow the progression of CALD. An OLE is ongoing (NCT03231878).

#### 3.4.5. Friedreich Ataxia (FRDA)

Multiple trials have been conducted in subjects with FRDA in recent years, and our search yielded seven publications since 2018, assessing six different drugs. Most trials used the Friedreich Ataxia Rating Scale (FARS) or modified FARS (mFARS), 9-hole peg test (9HPT), and 25 or 8 min walk tests to assess the efficacies of the drugs. However, none of these tests are specific to neuropathy progression. For example, the mFARS omits the peripheral nervous system subscore section of the FARS. Three drugs, luvadaxistat [81], (+)-epicatechin [80], and IFN-γ1b [78] did not demonstrate efficacy in terms of neurological outcomes. EPI-743 treatment was associated with a statistically significant improvement in neurological function and disease progression relative to a natural history cohort and also showed clinically meaningful improvement in the FARS-neuro at 6 months [79]. RT001 improved peak workload relative to baseline in a double-blind phase 1/2 study, and there was not a significant difference in FARS-neuro scores between the drug and comparator over a short period of 28 days [84]. Omaveloxolone statistically improved mFARS over placebo in a 12-week, dose-ranging study and in an international, double-blind, randomized, placebo-controlled, phase 2 trial conducted on 103 patients with a daily dose of 150 mg [82,83]. This drug has been approved by the FDA in 2023 but its efficacy on neuropathy is unknown.

#### 3.4.6. Fragile-X Associated Tremor/Ataxia Syndrome (FXTAS)

An open-label phase 2 trial was conducted on 10 FXTAS patients to assess the safety and efficacy of citicoline on motor and cognitive functions over a 1-year treatment period. There were no significant changes in FXTAS rating scale scores in treated subjects, although worsening was expected in this population. Moreover, most of the secondary outcome measures remained stable, and citicoline was well tolerated [93]. These findings suggest that citicoline may stabilize disease progression, but a larger study will be necessary to confirm these results.

### 3.5. Treatments for Conditions Where Neuropathy Is Inconsistent and/or Subclinical

#### 3.5.1. Acid Sphingomyelinase Deficiency (ASMD)

ASMD, also known as Niemann–Pick disease, is a lysosomal storage disease. Olipudase alfa is a human recombinant acid sphingomyelinase. IV administration is used in non-central nervous system manifestations as it does not cross the blood–brain barrier. Two open-label studies assessed this therapy in ASMD patients. In one study, five adults were treated for 30 months [92], and the other enrolled 20 paediatric patients with chronic ASMD [91]. Both studies showed a reduction in biomarkers and benefits in non-neurological parameters; however, neuropathy was not assessed. No adult patients developed antidrug antibodies, but antidrug antibodies were detected in eight paediatric patients. None tested positive for neutralizing antibodies that would interfere with enzyme uptake into cells, although one tested transiently positive for the inhibition of enzyme catalytic activity. One of the patients had an anaphylactic reaction but continued with treatment after desensitization. Further studies are required to evaluate the effects of olipudase alfa on neuropathy.

#### 3.5.2. Mitochondrial Encephalomyopathy with Lactic Acidosis and Stroke-like Episodes (MELAS)

Three peer-reviewed publications were found that describe the testing of amino acids L-arginine [67], taurine [69], and glutamine [68] in subjects with MELAS. None of these studies assessed neuropathy as a primary or secondary endpoint. Neither of the two open-label phase 3 studies conducted in Japan that evaluated taurine and L-arginine demonstrated improvement in treated subjects based on the Japanese Mitochondrial Disorder Rating Scale, but the frequencies of stroke-like episodes were decreased by treatment, and both amino acids had good safety profiles. In a study of the effects of glutamine, Guerrero-Moline et al. [68] showed that high-dose oral supplementation resulted in a decrease in CSF glutamate levels and an increase in glutamine levels in subjects with MELAS. Thus, L-arginine, taurine, and glutamine have potential and should be further evaluated.

#### 3.5.3. Gaucher Disease Type 1 (GD1)

Although GD1 is usually considered to be non-neuronopathic, there is growing evidence that peripheral nervous system manifestations are part of the clinical spectrum of this disease [14,118]. However, the aetiology of polyneuropathy associated with GD1 is unclear. It is not known if the imbalance in calcium homeostasis seen in the central nervous system of certain patients with GD1 [119] is also to blame for peripheral nerve injuries. Therefore, no biomarkers of peripheral nervous system involvement in GD1 have been validated.

Several therapies have been approved for the treatment of GD1. Three are ERTs: the recombinant β-glucocerebrosidases imiglucerase, velaglucerase alfa, and taliglucerase alfa. Another approach is substrate reduction therapy with glucosylceramide synthase inhibitors miglustat and eliglustat [120]. None of the identified trials assessed neuropathy as an outcome. In the phase 3 placebo-controlled, double-blind ENGAGE trial, eliglustat led to a reduction in several biomarkers, including median chitotriosidase, glucosylceramide (the primary sphingolipid that accumulates in GD1), glucosylsphingosine (a sphingolipid that is a highly specific validated biomarker of GD1), 4-monosialodihexosylganglioside (GM3, a precursor for more complex gangliosides), and macrophage inflammatory protein MIP-1β, which is a marker of metabolic inflammation [70]. Similar findings were reported after 8 years in previously untreated adults with GD1 who completed an open-label, phase 2 trial of eliglustat [71]. Further trials are required to test the efficacy of these drugs on neuropathy. It will be important to determine whether there is a correlation between neuropathy and any of the biomarkers, as this could help reveal the underlying mechanism of peripheral nervous system involvement in GD1.

#### 3.5.4. Bile Acid Synthesis Disorder (BASD)

Cholic acid and chenodeoxycholic acid, depending on the subtype of BASD, downregulate bile acid synthesis and are used clinically to treat BASD. The efficacy of cholic acid was demonstrated in a phase 3 open-label continuation study on 53 paediatric patients [85]. This study confirmed that cholic acid downregulates the production of atypical bile acids and improves liver biochemistries and growth in patients with BASD. Interestingly, one patient discontinued the study because of peripheral neuropathy that was reported as a treatment-emergent adverse event. It is possible that this neuropathy was a natural history progression. Further studies are warranted to evaluate the potential benefit of cholic acid on neuropathy in BASD.

## 4. Discussion

### 4.1. Limitations of This Analysis

Our search was limited to studies with results published after 1 January 2018. For this reason, several clinically validated therapies for inherited neuropathies (e.g., vitamin E for AVED, riboflavin for Brown–Vialetto–Van–Laere, diet to limit pristanic acid for Refsum disease, etc.) were not captured in our search (but are well described by Fernandez-Eulate et al. [2]), nor were unsuccessful trials prior to 2018 (e.g., ascorbic acid, progesterone antagonists/modulators in CMT1A) [8]. Similarly, suspended studies (e.g., the gene therapy scAAV1.tMCK.NTF3 for CMT1A; NCT03520751) were not considered. Finally, we did not include preclinical studies or recruiting trials, thereby missing potential future therapeutic approaches (e.g., gene therapies for Charcot–Marie–Tooth type 4J and X, AT007 for sorbitol dehydrogenase deficiency patients).

### 4.2. Added Value of This Analysis

Inherited neuropathies are challenging to diagnose and treat. Here, we provided an up-to-date list of conditions that may have neuropathy as a clinical feature, highlighting those that are treatable or for which treatment could soon be available.

The list of genes causative of neuropathy continues to grow, and whilst it is established that certain conditions (e.g., Friedreich ataxia) are associated with neuropathy, the latter can be a very minor and inconstant aspect of an inherited condition, making it difficult to confirm the association of neuropathy with a particular disease, especially in small cohorts of patients. This review identified neuropathies by beginning with gene panels used for the diagnosis of inherited neuropathies in the UK, France, and the USA; this list was extended by the authors and through searches of published data [1,2,3,14] and revised by two experts. Although it may not be exhaustive, the list provided here includes genes not described in recent publications and highlights the inconsistencies among published data and the different gene panels used for this analysis. Indeed, only a few genes were consistently found in all three panels, and some genes mentioned in the literature were not found in any of these three panels (Appendix A).

In addition to providing an updated list of conditions associated with inherited neuropathies, this review summarized data from clinical trials conducted in the last 5 years, evaluating therapies for these specific conditions. This analysis allowed us to identify 28 studies evaluating the effects of drugs or dietary interventions on neuropathy. Among these, 14 different drugs were assessed in nine inherited neuropathies. Nine additional therapies were identified that were not included in the systematic review published by Jennings et al. in 2021 [7], attesting to the increased interest in this field over recent years. Our search yielded several disease-modifying drugs, including small molecules, nucleic acid-based drugs, and gene therapy, especially in patients with ATTRv-PN and MLD. Some trials looked promising (e.g., L-serine for HSAN1 and PTX3003 for CMT1A) and will require close monitoring in the future, whereas others showed relatively stable nerve conduction studies upon treatment (e.g., DHA for SCA 38, rhASA for MLD) and warrant longer follow-ups and/or comparisons to natural history cohorts to assess the effects on neuropathy. Finally, some treatments of symptoms were beneficial (e.g., carbidopa in familial dysautonomia).

Although not designed to evaluate neuropathy as an endpoint, several therapeutics were demonstrated to cause changes in biomarkers thought to be causal of neuropathies (i.e., NTLA-2001 and eplontersen for ATTRv-PN, givosiran for AIP, and ERT, EET, and SRT for Fabry disease). These therapeutics should be tested with a focus on neuropathy in further trials. Other therapeutics caused changes in biomarkers but the association with neuropathy is unknown (i.e., taurine and glutamine for MELAS, SRT and ERT for Gaucher disease, several trials in FRDA, cholic acid in BASD, leriglitazone in X-ALD, and olipudase alfa in ASMD). Finally, improvements in neurological assessments were observed for patients with FRDA treated with EPI-743 and omaveloxolone and for patients with X-ALD treated with leriglitazone, but further investigations are needed in terms of efficacy on neuropathy, especially since omaveloxolone has recently been approved by the FDA.

In addition, this research highlighted two general findings. Firstly, most clinical trials have been conducted with subjects diagnosed with complex disorders with a possible neuropathology component rather than pure hereditary neuropathies. As neuropathy is only one of a number of symptoms that can be experienced by these subjects, fewer than 50% of studies monitored nerve conduction or employed specific neuropathy scales to assess treatment efficacy. Secondly, among clinical trials with neuropathy as an outcome measure, various clinical scales or scoring systems were used, which prevented comparisons between studies. Moreover, some scoring systems, although validated for use in the evaluation of the progression of neuropathy, may not be appropriate in all circumstances. For example, heart rate to deep breathing from the modified Neuropathy Impairment Score +7 scale, which is validated for use in neuropathies involving autonomic dysfunction [121], was not performed in the NEURO-TTR trial evaluating inotersen, because most ATTRv-PN patients included in this trial had active pacing or atrial fibrillation. Therefore, other endpoints must be used to assess autonomic dysfunction in such subjects. Additional limitations are that the scoring systems are subjective and cannot reliably detect subtle motor changes, especially in small cohorts.

Therefore, it will be important to assess neuropathy as an outcome in further trials, aiming to support an association of various complex conditions with neuropathies amongst larger cohorts. In addition, it will be critical to develop objective and reliable methods for this analysis. Research is ongoing to evaluate disease progression in subjects with peripheral neuropathies, including the use of wearable technologies [122,123], nerve sonography [124], intramuscular fat accumulation demonstrated by MRI imaging of the lower limbs [125], elevated plasma neurofilaments light chain concentration [126], and changes in the motor unit index (MUNIX) (NCT03715283). Associated with systematic nerve conduction studies, this could allow a better characterization of the type of neuropathy, their prevalence, and their potential correlation with readily monitored biomarkers to lead to a better understanding of the underlying physiopathological mechanisms of neuropathy and more effective treatment.

## Figures and Tables

**Table 1:** Summary of clinical trial results in which neuropathy was assessed as a primary or secondary outcome.

Disease	Compound	Dosing and Administration	Mechanism of Action	Clinical Trial Identifier	Biological/Histological Outcome	Clinical Outcome	Electrophysiological Outcome	Current Status
Hereditary transthyretin amyloidosis polyneuropathy (ATTRv-PN)	Tafamidis/ FX-1006	20 mg, 1x/d, O	TTR stabilizer	NCT00925002 (phase 3 LTE) [18,19], NCT00409175 (phase 1/2), NCT00630864 (phase 2) [20].	/	- Mean (95% CI) change from baseline in NIS-LL of 5.3 (1.6, 9.1) points after 5.5 years vs. NHx progression of 4 points/year among patients with a mild neuropathy at treatment start (NIS-LL ≤ 10). - Similar baseline-adjusted mean change in NIS-LL at month 12 between Val30Met and non-Val30Met. - Approximately 85% of Val30Met and 75% of non-Val30Met patients were alive at 9 and 8 years, respectively.	/	- EMA approval in 2011 for patients with stage 1 polyneuropathy (brand name VYNDAQEL, Pfizer). - Not approved by FDA for ATTRv-PN but approval was granted in 2019 for use at a higher dose in ATTR-associated cardiomyopathy [21].
	Patisiran/ALN-18328	0.3 mg/kg 1x/3 w, IV	siRNA targeting *TTR* mRNA	NCT01960348 (phase 3; APOLLO) [22,23,24], NCT01961921 (phase 2 OLE) [25,26], NCT03862807 (phase 3b) [27]	- Reduction in serum TTR levels. - Exploratory analyses demonstrated improvement in nerve fibre density with corresponding - reductions in amyloid burden observed in skin biopsies over 24 months.	- Significant improvement in the mNIS + 7, Norfolk QOL-DN score, gait speed, mBMI (least-squares mean change from baseline *p* < 0.001), and COMPASS-31 score (*p* = 0.0008) with clinical effects observed 9 months after initiation.	/	- FDA approval in 2018 for ATTRv-PN of any stage and EMA approval in 2018 for the treatment of ATTRv-PN in adults with stage 1 or 2 polyneuropathy (brand name Onpattro, Alnylam Pharmaceuticals) [28].
	Vutrisiran/ ALN-TTRSC02	25 mg, 1x/3 monts, SC	siRNA targeting *TTR* mRNA	NCT03759379 (phase 3; HELIOS-A study) [29]	- TTR reduction was similar to patisiran.	- Significant change from baseline in mNIS + 7 at 9 months (*p* = 3.54 × 10^−12^) and significant improvements vs. placebo-arm of APOLLO in Norfolk QOL-DN, 10 m walk test, mBMI, and R-ODS (all at 18 months).	/	- FDA approval in 2022 for adult patients with ATTRv-PN and EMA approval in 2022 for adult patients with stage 1 or stage 2 polyneuropathy (brand name Amvuttra, Alnylam Pharmaceuticals) [30].
	Inotersen/ IONIS-TTRRX or ISIS 420915	300 mg 1x/w, SC	Antisense oligonucleotide targeting *TTR* mRNA	NCT01737398 (phase 3; NEURO-TTR) [31,32,33,34], NCT02175004 (phase 3 OLE) [35,36,37,38]	- Reduction in serum TTR levels.	- mNIS + 7 and Norfolk QOL-DN score both favoured inotersen over placebo at week 66 (*p* < 0.001), independent of disease stage, mutation type, or the presence of cardiomyopathy. - Improvements sustained at week 156 of the OLE.	- Individual assessment of ulnar CMAP significantly favoured inotersen vs. placebo (*p* = 0.015)	- FDA approval in 2018 for ATTRv-PN of any stage, and EMA approval in 2018 for patients with stage 1 or 2 polyneuropathy (brand name Tegsedi, Akcea Therapeutics) [39].
Spinocerebellar ataxia type 38 (SCA 38)	Docosahexaenoic acid	600 mg/d, O	Replenish low levels of serum docosahexaenoic acid caused by ELOVL5 gene mutation	NCT03109626 [40]	- Slight but not significant increase in total serum DHA compared to baseline (*p* = 0.41) at 104 weeks. - No significant reduction in ELOVL5 expression in blood (*p* = 0.75) at 104 weeks.	- Significant reduction (i.e., improvement) in SARA score and ICARS score (posture, gait, kinetics) compared to baseline (*p* = 0.013 and *p* < 0.001, respectively) at 104 weeks.	- Motor and sensory conduction velocities did not significantly worsen.	- Further investigations needed.
Familial dysautonomia	Carbidopa	300 or 600 mg/d, O	Selective dopa-decarboxylase inhibitor that suppresses catecholamine production outside the brain	NCT02553265 (phase 2) [41]	- Significant reduction in urinary norepinephrine levels (*p* = 0.0075) at both doses.	- Significant effect on daytime systolic blood pressure variability compared to placebo (*p* = 0.0013) at both doses and reduction in the systolic blood pressure peaks (*p* = 0.0015).	/	- Provides class Ib evidence that carbidopa can reduce blood pressure variability in patients with congenital afferent baroreflex failure (symptomatic), but further investigations needed.
Hereditary sensory and autonomic neuropathy (HSAN1)	L-serine	400 mg/kg/d, O	Provides normal substrate of enzyme serine palmitoyltransferase	NCT01733407 (phase 1/2) [42]	- 1-deoxySL levels declined significantly (*p* < 0.001). - Significant change at 1 year in epidermal nerve fibre density at distal site.	- Quantitative improvement in the CMTNS compared to placebo after 2 years (*p* = 0.09).	- No treatment effects were detected in nerve conduction studies.	- Further investigations needed.
Charcot– Marie–Tooth 1A (CMT1A)	Combination of baclofen, naltrexone and sorbitol/ PXT3003	5 mL, 2x/d, O	Downregulation of PMP22 overexpression	NCT02579759 (phase 3) [43]	/	- Significant improvement in the ONLS score (*p* = 0.008) and 10MWT (*p* = 0.016) in the high-dose group.	- No statistically significant change in DML, MCV, CMAP, and radial SNAP.	- Open-label continuation study (NCT03023540) ongoing.
Metachromatic Leuko-dystrophy (MLD)	rhASA; HGT-1111/ Metazym	100 or 200 U/kg, EOW, IV	Enzyme replacement therapy	NCT00681811 (phase 1/2 OLE; Study-049) [44]	- Mean CSF sulfatide levels decreased from baseline non-significantly (*p* = 0.1363). - No statistically significant change in morphometric measurements of the sural nerves. - Sural nerve sulfatide and lysosulfatide levels remained relatively stable with considerable interindividual variability.	- Decrease in mean GMFM-88 total score, indicating declining motor function.	- No statistically significant changes in SNAP, CMAP, DML, or NCV.	- Study terminated after 24-month extension.
	rhASA/HGT-1110	10, 30, or 100 mg or 100 mg manufactured using a revised process, EOW, IT	Enzyme replacement therapy	NCT01510028 (phase 1) [45]	- CSF sulfatide and lysosulfatide levels fell to within normal ranges in both 100 mg cohorts.	- General decline in GMFM-88 score (tendency towards a less pronounced decline in patients receiving 100 mg than in the other cohorts).	- Stable nerve function.	- Extension study (HGT-MLD-071; NCT01887938) ongoing.
	Atidarsagene autotemcel (arsa-cel)/ HSPC-GT	1 delivery	Gene therapy	NCT01560182 (phase 1/2) [46]	- ARSA activity in PBMCs significantly increased (*p* < 0.0001) in both late-infantile and early-juvenile populations. - Mean ARSA activity in cerebrospinal fluid reached normal levels.	- Significant differences in GMFM-88 between treated patients and age-matched and disease subtype-matched NHx cohorts (late-infantile MLD, 66% [95% CI 48.9–82.3], and early-juvenile MLD, 42% [12.3–71.8].	- Significant differences in NCV between treated patients with late-infantile MLD and NHx patients at year 2 (*p* = 0.004) and year 3 (*p* = 0.010).	- Approved by EMA in 2020 and in the UK in 2021 (brand name Libmeldy, Orchard Therapeutics) [47]. - Under investigation in the USA. - Ongoing study in the late-juvenile variant of MLD (NCT04283227).
X-adrenoleuko- dystrophy (X-ALD)	Combination of α-tocopherol (also known as vitamin E), N-acetyl-cysteine (NAC), and α-lipoic acid (LA).	Dose A: NAC (800 mg), LA (300 mg), and α-tocopherol (150 IU) or dose B: NAC (2400 mg), LA (600 mg), and α-tocopherol (300 IU)	Antioxidant	NCT01495260 (phase 2) [48]	- Decrease in oxidative damage markers, pro-inflammatory markers, and markers of immune system activation (neopterin). - Increase in anti-inflammatory plasma adiponectin.	- Significant improvement (*p* = 0.03) in the distance walked in the 6MWT and stable EDSS scores.	- LEP signals increased in 8/13 subjects. - Significant decrease in the CMCT of MEP in both legs. - SEP unchanged. - No difference in nerve conduction studies.	- The mild clinical improvement was not explicitly linked to neuropathy. Further investigations needed.
Spinal and bulbar muscular atrophy (SBMA)	Mexiletine	100 mg, 3x/d, O	Sodium- channel inhibitor	UMIN000020426 (phase 2; MEXPRESS) [49]	/	- ALSFRS-R improved non- significantly. - Significant improvements were shown in tongue pressure (*p* = 0.023) and 10-s grip and release test under cold exposure (*p* = 0.002).	- No significant difference in distal latencies at room temperature and under cold exposure conditions between mexiletine group and placebo (*p* = 0.843).	- Potential as a symptomatic therapy, but further investigations needed.
Adult polyglucosan body disease (APBD)	Triheptanoin	1 mg/kg, 3–4x/d, O	Anaplerotic properties	NCT00947960 (phase 2) [50]	/	- No statistically significant change in the 6MWT vs. placebo. Motion capture gait analysis, gait quality, and stair climbing showed no consistent direction of change.	- Two mildly disabled patients had stable nerve conduction studies 2 and 4 years after triheptanoin discontinuation.	- Further investigations needed.

The backslash indicates that the analysis was not performed. Abbreviations: O, orally; TTR, transthyretin; NIS-LL, Neuropathy Impairment Score in the Lower Limbs; NHx, natural history cohorts; EMA, European Medicines Agency; FDA, US Food and Drug Administration; W, week; IV, intravenous; siRNA, small interfering RNA; mNIS + 7, modified Neuropathy Impairment Score + 7; Norfolk QOL-DN, Norfolk Quality of Life-Diabetic Neuropathy; mBMI, modified body mass index; COMPASS-31, Composite Autonomic Symptom Score-31; SC, subcutaneously; R-ODS, Rasch-built Overall Disability Scale; OLE, open-label extension; CMAP, compound muscle action potential; SARA, Scale for the Assessment and Rating of Ataxia; ICARS, International Cooperative Ataxia Rating Scale; 1-deoxySL, 1-deoxysphingolipids; CMTNS, Charcot–Marie–Tooth neuropathy score; PMP22, Peripheral Myelin Protein 22; ONLS, Overall Neuropathy Limitations Scale; 10MWT, 10 m walk test; DML, distal motor latency; MCV, motor nerve conduction velocities; CMAP, compound muscle action potential; SNAP, sensory nerve action potential; CSF, cerebral spinal fluid; GMFM, Gross Motor Function Measure; ARSA, arylsulfatase A; PBMC, peripheral blood mononuclear cells; NCV, nerve conduction velocities; IT, intrathecal; 6MWT, 6 min walk test; EDSS, Expanded Disability Status Scale; LEP, laser-evoked potential; MEP, motor-evoked potential; SEP, sensory-evoked potential; ALSFRS-R, Revised Amyotrophic Lateral Sclerosis Functional Rating Scale.

**Table 2:** Summary of clinical trial results in which neuropathy was not assessed as a primary or secondary outcome.

Disease	Compound	Dosing and Administration	Mechanism of Action	Clinical Trial Identifier	Biomarker Outcome	Neurological Clinical Outcome	Current Status
ATTRv-PN	Eplontersen/ AKCEA-TTR-LRx/ ION-682884	45 mg, 1x/m, SC	Antisense oligonucleotide targeting *TTR* mRNA	NCT03728634 (phase 1), NCT04302064 (phase 1) [51]	- Reduction in serum TTR levels.	/	- Phase 3 trials NCT04136184 and NCT04136171 ongoing. - Ionis announced FDA acceptance of New Drug Application in 2023 [52].
	Acoramidis/ AG10	800 mg, 2x/d, O	TTR stabilizer	NCT03860935 (phase 3; ATTRibute-CM Study) [53]	- Increased serum TTR levels relative to placebo (*p* < 0.01), consistent with stabilization of TTR.	- Primary endpoint at month 12 (change in 6MWT) was not achieved (*p* = 0.76).	- 30-month phase B in ATTRv-CM ongoing (NCT03860935) and open-label study (NCT04988386) to follow. - Trial assessing the treatment in ATTRv-PN terminated by the sponsor.
	NTLA-2001	0.1 mg/kg or 0.3 mg/kg, single dose, IV	CRISPR-Cas9- mediated editing of *TTR*	NCT04601051 (phase 1) [54]	- Reduction in serum TTR levels at day 28 by 52% in the 0.1 mg/kg group and 87% in the 0.3 mg/kg group.	/	- Phase 1 study ongoing.
	Tolcapone/ SOM0226	100 mg 3x/d, O	TTR stabilizer	NCT02191826 (phase 2) [55]	- Serum TTR tetramer increase value of at least 20% reached 2 h after dosing.	/	- FDA approval as an adjunct to levodopa/carbidopa for Parkinson’s disease (brand name Tasmar, Roche Pharmaceuticals). - Under development by Corino Therapeutics as a repurposed drug to treat ATTRv-PN and other forms of ATTR, including leptomeningeal amyloidosis [56].
Fabry Disease	Migalastat hydrochloride	150 mg, EOD, O	Chaperone to enhance endogenous alpha-galactosidase A activity	NCT03362164 (HEALFABRY) [57], NCT02194985 (phase 3 OLE; AT1001-042) [58,59], NCT00925301 (phase 3; FACETS) [60]	- Increased alpha-galactosidase-A activity in leucocytes. - Plasma Lyso-Gb3 decreased.	/	- Approved for amenable *GLA* mutations by FDA in 2004 and EMA in 2006 (brand name Galafold; Amicus Therapeutics). - Efficacy on neuropathy unknown.
	Venglustat	15 mg, 1x/d, O	Substrate reduction therapy: inhibits glucosylceramide synthase-mediated conversion of ceramide to glucosylceramide-1 (GL-1), which is a precursor of Gb3	NCT02489344 (phase 2 OLE) [61]	- Proximal markers (plasma GL-1 and GM3) reduced at week 2 (*p* = 0.0001 and *p* = 0.0007, respectively) and more distal markers (plasma Gb-3 and lyso- Gb3) reduced at week 26 (*p* < 0.0001 and *p* = 0.0036, respectively).	- Bodily pain (SF-36) improved at week 26 in a majority of patients; results were not consistent at week 156.	- Randomized controlled phase 3 clinical trials ongoing (NCT05206773 and NCT05280548).
	Agalsidase beta	0.5 mg/kg/2 w or 1 mg/kg/4 w, IV	Enzyme replacement therapy	NCT00701415 (phase 3b) [62]	- Clearance of Gb-3 from SSCE and podocytes not consistent. - Urine Gb-3 levels decreased but were still above normal in 59.3% of patients after 5 years. - Plasma Gb3 concentrations normalized starting at month 3 (*p* < 0.0001).	/	- FDA approval since 2003 (brand name Fabrazyme, Sanofi Genzyme). - This study supports the approved dose of 1 mg/kg/2 w.
	Agalsidase beta/ ISU303	1 mg/kg/2 w, IV	Enzyme replacement therapy	Open-label * (phase 2) [63].	- Significant decrease in plasma and urine Gb3 (*p* = 0.005 and *p* = 0.017, respectively). - No significant decrease in plasma and urine lyso-Gb3 (*p* = 0.13 and *p* = 1.00, respectively).	- Trend toward improvement in Short-Form McGill Pain Questionnaire (*p* =0.19) and subject symptoms diary (*p* = 0.63). - SF-36 Health Status Survey was stationary (*p* = 0.84).	- Potential alternative agent to Fabrazyme, developed by ISU Abxis.
	Pegunigalsidase alfa	Different doses, EOW, IV	PEGylated ERT	NCT01769001 (phase 1/2) [64]	- Reductions in plasma Gb3 and lyso-Gb3 levels. - Mean decrease in peritubular capillary Gb3 content of 67.8% ± 8.9%.	/	- Peer-review publication of the 60-month extension study (NCT01981720) awaited.
Spino-cerebellar ataxia type 2 (SCA2)	Riluzole	50 mg, 2x/d, O	Neuroprotective action resulting from enhanced synaptic reuptake of glutamate and decreased release	NCT03347344 (phase 3; ATRIL) [65]	/	- SARA score worsened. - No change in INAS score.	- Study did not demonstrate efficacy in this population.
	Human recombinant erythropoietin (NeuroEPO)	1 mg, 3x/w	Neurotrophic factor	Cuban Public Registry of Clinical Trials: RPCEC00000187-Sp [66]	/	- SCAFI ≥ 0.75 was marginally higher (*p* = 0.092) in the NeuroEPO group (N = 6, 46.2%) than in placebo (N = 2, 12.5%). - Intergroup differences in the mean delta SARA scores were not significant.	- A phase III trial with a 1-year duration in a larger population of Cuban SCA2 patients sampled with a stratified randomization method is planned.
Mitochondrial encephalopathy, lactic acidosis, and stroke-like episodes (MELAS)	L-arginine	0.3–0.5 g/kg/day, O plus 0.5 g/kg IV, if ictus	NO-mediated vasodilatation	OL-MELAS study integrated the pooled data from JMACTRIIA00023 (phase 3) and JMACTR-IIA00025 (phase 3) [67]	/	- JMDRS scores in Section 1 and Section 2 (activities of daily living and motor, respectively) worsened at the end of the 7-year follow-up (7.29 ± 8.77 and 24.2 ± 11.7, respectively).	- This study supports the administration of oral and intravenous L-arginine as it decreases the frequency of stroke-like episodes but effects on neuropathy are unknown.
	Glutamine	6–18 g/24 h, O	Reduce excitotoxic glutamate levels	NCT04948138 [68]	- CSF glutamate levels were significantly reduced (*p* < 0.001) - CSF glutamine levels were significantly increased (*p* = 0.002). - Taurine CSF levels were significantly decreased (*p* = 0.043).	/	- Future studies are warranted to determine the potential therapeutic effect of such supplementation.
	Taurine	9–12 g/d, O	Restores the taurine modification defect in mutant mitochondrial tRNA^Leu(UUR)^ and ameliorates mitochondrial dysfunction	UMIN000011908 (phase 3) [69]	- Rate of taurine modification of mitochondrial tRNA^Leu(UUR)^ from peripheral blood leucocytes was significantly increased in 5/9 patients (*p* < 0.05).	- No significant change in JMDRS section 1 (*p* = 0.7969) or 2 (*p* = 0.8125).	- Treatment decreases the frequency of stroke-like episodes in previously L-arginine-treated patients. - Synergy of taurine and L-arginine must be evaluated. - Effect on neuropathy unknown.
Gaucher Disease type 1 (GD1)	Eliglustat	50 or 100 mg, 2x/d, O	Substrate reduction therapy	NCT00891202 (phase 3; ENGAGE OLE) [70], NCT00358150 (phase 2 OLE) [71]	- Decrease in glucosylceramide and glucosylsphingosine levels, GM3, MIP-1β activity and chitotriosidase activity.	/	- Approved by FDA in 2014 for adults with GD1 who are CYP2D6 extensive, intermediate, or poor metabolizers (brand name Cerdelga, Sanofi Genzyme). - Effect on neuropathy unknown.
	Taliglucerase alfa	30 or 60 U/kg, EOW, IV	Enzyme replacement therapy	NCT01411228 (phase 3; PB-06-006) [72], NCT00962260; phase 3; PB-06-004) [73]	- Decrease in chitotriosidase (−72.7% and −84.4% at 30 and 60 U/kg, respectively).	/	- Approved by FDA in 2012 for paediatric and adult GD1 patients (brand name Elelyso, Pfizer and Protalix BioTherapeutics). - Effect on neuropathy unknown.
Acute intermittent porphyria (AIP)	Givosiran	1.25–2.5 mg/kg, 1x/month, SC	siRNA targeting *ALAS1* mRNA	NCT03338816 (phase 3 OLE; ENVISION) [74,75,76], NCT02452372 (phase 1) [77]	- Urinary ALA and PBG lowered. - ALAS1 mRNA levels reduced.	- Annualized attack rate (AAR), hemin use, daily worst pain reduced.	- Givosiran (brand name GIVLAARI, Alnylam Pharmaceuticals) was approved in 2019 by the FDA for adult AHP patients and by the EMA for patients aged 12 years and older experiencing more than 4 attacks in a 12-month period.
Friedreich ataxia (FRDA)	IFN-γ 1b/ACTIMMUNE	10, 25, 50, 100 µg/m^2^, 3x/w, SC	Increases frataxin mRNA and protein levels in a variety of cell types	NCT02593773 (phase 3) [78]	- Frataxin levels were identical in both whole blood and buccal cell isolates between IFN and placebo groups at the end of the double-blind period, but buccal cell frataxin levels increased during the OLE period.	- No difference between the active agent and placebo groups for FARS/mFARS, T25FW, and 9- HPT, but modest stabilization compared to NHx during an OLE period.	- Longer studies are warranted to evaluate benefit.
	EPI-743/alpha- tocotrienolol quinone/vatiquinone	200 or 400 mg, 3x/d, O	Targets oxidoreductase enzymes essential for redox control of metabolism	NCT01728064 (phase 2) [79]	/	- No statistically significant differences in FARS-neuro (*p* = 0.12852), 9HPT (*p* = 0.69654), or 25FWT-1 (*p* = 0.88076) compared to baseline but improvement in FARS-neuro compared to NHx patients at 24 months (*p* < 0.001).	- Further studies warranted.
	(+)-Epicatechin	Escalation of total dose from 75 to 150 mg/d, O	Mitochondrial biogenesis	NCT02660112 (phase 2) [80]	- Follistatin levels increased significantly. - Myostatin levels did not change.	- No significant improvements in FARS/mFARS scores, 8 m walk, or 9-peg hole test.	- Longer studies are warranted to evaluate benefit.
	Luvadaxistat/ TAK-831/ NBI-1065844	75 mg or 300 mg, 2x/d, O	Inhibitor of D-amino acid oxidase, which mediates D-serine breakdown.	NCT03214588 (phase 2) [81]	- Increase in mean plasma D-serine from baseline in both groups.	- No significant change from baseline for mFARS, 9HPT, or 25FWT-1.	- Longer studies are warranted to evaluate benefit.
	Omaveloxo- lone	150, 160, or 300 mg/d, O	Nrf2 activator	NCT02255435 (phase 2) [82,83]	/	- mFARS improved compared to baseline (*p* = 0.014 and *p* = 0.0001 for low- and high-dose groups, respectively). - No significant changes in the peak workload in maximal exercise testing, 9HPT, or 25FWT.	- FDA approval in February 2023 (brand name Skyclarys, Reata Pharmaceuticals) - Effects on neuropathy unknown.
	RT001/Stabilized Linoleic Acid (LA, ethyl ester)	1.8 or 9 g/d, O	Inhibits lipid peroxidation	NCT04102501 (phase 1/2) [84]	/	- Statistically significant improvement in peak workload. - Improvement in FARS-neuro without reaching statistical significance (*p* = 0.09 excluding a single subject in placebo cohort who experienced a very high improvement).	- Longer studies are warranted to evaluate benefit.
Bile acid synthesis disorder (BASD)	Cholic acid	10–15 mg/kg/d, O	Downregulates bile acid synthesis, thereby reducing the production of intermediates that lead to atypical bile acids	NCT01438411 (phase 3) [85]	- Statistically significant improvements in urine bile acid FAB-MS scores. - Decrease in serum ALT and AST. - Liver biopsies showed either stable findings or histologic improvement in all parameters except bridging fibrosis.	/	- Approved by the FDA in 2015 (brand name CHOLBAM, Travere Therapeutics). - Effectiveness on extrahepatic manifestations has not been established [86].
Ataxia- Telangiectasia (A-T)	Betamethasone	0.02 mg/kg/d, O	Not described	UMIN000004109 [87]	/	- Transient improvement in SARA and A-T NEST scores at 6 months not sustained.	- Transient positive effect not explicitly linked to neuropathy.
	Intra-Erythrocyte Dexamethasone Sodium Phosphate/EDS-EP	Dose range of 5–10 mg or 14–22 mg/infusion, 1/month, IV	Ex-vivo encapsulation of the molecule into patient’s red blood cells, which are then re-infused, allowing a slower release	NCT02770807 (phase 3; ATTeST) [88]	/	- Statistically significant differences vs. placebo for mICARS (*p* = 0.020) and RmICARS (*p* = 0.025).	- Study completed, peer-reviewed results awaited. - FDA and EU Marketing Authorization filings expected in 2023.
X-adreno-leukodystrophy (X-ALD)	Leriglitazone/ MIN-102	150 mg starting dose then adjusted to achieve plasma concentrations of 200 μg·h/mL/d, O	Selective PPARγ agonist	NCT03231878 (phase 2/3; ADVANCE) [89]	- Higher change from baseline in adiponectin compared to placebo. - Reductions in concentration of nfL, MMP, and pro-inflammatory markers compared to placebo.	- No statistical difference in the total distance walked in the 6MWT compared to placebo (*p* = 0.91). - In post hoc subgroup analyses, patients with early-stage disease had numerically less decline in 6MWT and EDSS scores at week 96 compared to placebo.	- Open-label extension trial (NCT03231878) ongoing.
Spinal and bulbar muscular atrophy (SBMA)	IGF-1 mimetic/ BVS857	0.06 mg/kg, 1x/w, IV	Stimulates Akt-mediated phosphorylation of the mutant AR, which enhances its clearance	NCT02024932 [90]	- No significant increase in Akt signalling in monocytes.	- No improvement in strength of the quadriceps or hamstring muscles or other functional muscle testing. - Significant difference in change in TMV compared to placebo (*p* = 0.02).	- Further investigations warranted.
Acid sphingomyelinase deficiency (ASMD)	Olipudase alfa	Dose escalation from 0.03 mg/kg to 3 mg/kg, 1x/2 W, IV	ERT	NCT02292654 (phase 1/2; ASCEND-Ped) [91], NCT02004704 [92]	- Decrease in plasma/dried-blood spot lyso-sphingomyelin levels and chitotriosidase activity.	/	- Further investigations warranted.
Fragile-X tremor and ataxia syndrome (FXTAS)	Citicoline	1000 mg, 1x/d, O	Endogenous nucleotide and intermediate in the biosynthesis of structural membrane phospholipid.	NCT0219710 (phase 2) [93]	/	- No significant change in the FXTAS-RS after 1 year (*p* = 0.18) in the 7 m i-TUG gait test, CDP balance testing with the Sensory Organization Test (SOT), or 9HPT.	- Available over the counter in the US.

The backslash indicates that the analysis was not performed. * No trial identifier available. Abbreviations: M, month; SC, subcutaneously; TTR, transthyretin; FDA, US Food and Drug Administration; d, day; O, orally; 6MWT, 6-minute walk test; ATTRv-CM, hereditary transthyretin amyloid cardiomyopathy; IV, intravenous; CRISPR-Cas9, clustered regularly interspaced short palindromic repeats and associated Cas9 endonuclease; EOD, every other day; lyso-Gb3, globotriaosylsphingosine; Gb3, globotriaosylceramide; GM3, monosialoganglioside; SF-36, 36-Item Short Form Survey; SSCE, superficial skin capillary endothelium; W, week; QOL, quality of life; SARA, Scale for the Assessment and Rating of Ataxia; INAS, Inventory of Non-Ataxia Signs; SCAFI, spinocerebellar ataxia functional index; NO, nitric oxide; JMDRS, Japanese mitochondrial disease-rating scale; CSF, cerebrospinal fluid; MIP-1β, macrophage inflammatory protein-1β; siRNA, small interfering RNA; ALA, aminolevulinic acid; PBG, porphobilinogen; ALAS1, delta-aminolevulinate synthase 1; AHP, acute hepatic porphyria; OLE; open-label extension; FARS, Friedreich Ataxia Rating Scale; mFARS, modified Friedreich Ataxia Rating Scale; T25FW, timed 25-foot walk; 9-HPT, 9-hole peg test; NHx, natural history cohorts; BID, bis in die; FAB-MS, fast atom bombardment mass spectrometry; ALT, alanine aminotransferase; AST, aspartate aminotransferase; AT-NEST, Ataxia-Telangiectasia Neurological Examination Scale Toolkit; ICARS, International Cooperative Ataxia Rating Scale; SPA, special protocol assessment; nfL, neurofilament; MMP, matrix metalloproteinase; EDSS, Expanded Disability Status Scale; AR, androgen receptor; TMV, thigh muscle volume; FXTAS-RS, Fragile X-Associated Tremor Ataxia Syndrome Rating Scale; TUG, timed up and go; CDP, computerized dynamic posturography.

## Appendix A

**Table A1:** List of inherited neuropathies and associated genes.

Classification	Disease and Synonyms		Gene	UK Panel	USA Panel	France Panel	Literature
Mechanism depending on the variant	Charcot–Marie–Tooth, also known as hereditary motor and sensory neuropathy, Dejerine-Sottas syndrome, and peroneal muscular atrophy		Several causative genes	X	X	X	[3,14]
	Hereditary sensory and autonomic neuropathy (HSAN), also known as hereditary sensory neuropathy (HSN)		Several causative genes	X	X	X	[3,14]
	Hereditary motor neuropathy (HMN)		Several causative genes	X	X	X	[3,14]
	Spinocerebellar ataxia (SCA) (1–38)		Several causative genes	X	X	X	[1,3,14,122]
	Hereditary spastic paraplegia (HSP), also known as familial spastic paraparesis (1, 2, 3A, 4, 5A, 6, 7, 9A/B, 10, 11, 12, 14, 15, 17, 20, 25, 26, 27, 28, 30, 31, 36, 38, 39, 43, 46, 47, 49, 55, 56, 57, 61, 76)		Several causative genes	X	X	X	[1,3,14]
	Porphyria	Coproporphyria	*CPOX*	X	X		
		Variegata	*PPOX*	X	X		
		Acute intermittent	*HMBS*	X	X		
		DOSS porphyria	*ALAD1*				[14]
Peroxisomal	Zellweger spectrum disorder		*PEX10, PXMP2*	X			
	Refsum disease		*PHYH, PEX7*	X	X		
	X-linked adrenoleukodystrophy (X-ALD)		*ABCD1*	X			
	Alpha-methylacyl-CoA racemase deficiency (AMACRD)		*AMACR*	X			
Lysosomal	GM2-gangliosidose AB, also known as Tay-Sachs disease and Sandhoff disease		*GM2A, HEXA, HEXB*	X			
	Metachromatic leukodystrophy (MLD)		*ARSA*	X	X		
	DEGS1 insufficiency		*DEGS1*	X			
	Krabbe disease, also known as globoid leukodystrophy		*GALC*	X	X		
	Fabry disease		*GLA*	X	X	X	
	Chediak–Higashi syndrome		*LYST*	X			
	Kanzaki, also known as Schindler type II		*NAGA*	X			
	Acid sphingomyelinase deficiency (ASMD), also known as Niemann–Pick A, B		*SMPD1*				[3,127,128]
	B-mannosidosis		*MANBA*				[3,14]
	Sialidosis type 1		*NEU1*				[129]
	Salla disease		*SLC17A*				[130]
	Acid ceramidase deficiency, also known as Farber disease		*ASAH1*				[131]
	Multiple sulfatase deficiency, also known as Austin disease		*SUMF1*				[132]
	Glucocerebrosidase deficiency, also known as Gaucher disease		*GBA*				[14,118]
	Galactosialidosis		*CTSA*				[1,14]
Mitochondrial	Mitochondrial neurogastrointestinal encephalopathy (MNGIE)		*RRM2B*	X			
			*POLG*	X	X	X	
			*TYMP*	X	X		
	Neuropathy, ataxia, and retinitis pigmentosa (NARP) syndrome		*MT-ATP6*	X			
	Mitochondrial encephalomyopathy lactic acidosis and stroke-like episodes (MELAS)		*MT-TL1*	X			
	Sensory ataxic neuropathy, dysarthria, and ophthalmoparesis (SANDO)		*POLG*	X	X	X	
			*TWNK* also known as *C10ORF2*		X	X	
	Myoclonic epilepsy associated with ragged red fibres (MERRF)		*MTTK*				[1,3,14]
	Infantile-onset spinocerebellar ataxia, ophthalmoplegia, hypotonia, ataxia, hypoacusis, and athetosis (IOSCA), also known as OHAHA syndrome		*TWNK* also known as *C10ORF2*		X	X	
	Leigh Syndrome		*SURF1*	X	X		
	Kearns–Sayre syndrome		*deletion mtDNA*				[1]
	Friedreich ataxia (FRDA)		*FXN*	X	X	X	
	Pyruvate carrier deficiency		*MPC1*		X		
	Pyruvate dehydrogenase complex deficiency		*PDHA1*	X			
	Trifunctional protein deficiency with myopathy and neuropathy (MTP), also known as LCHAD deficiency		*HADHA*/*HADHB*	X	X		
	Multiple Acyl-Coa Dehydrogenase Deficiency (MADD)		*ETFHD*				[2]
	Infantile-onset multisystem neurologic, endocrine, and pancreatic disease (IMNEPD)		*PTRH2*		X		
	Mitochondrial complex IV deficiency nuclear type 2 (Mc4dn2), also known as cardioencephalomyopathy, fatal infantile, due to cytochrome c oxidase deficiency (CEMCOX1)		*SCO2*		X		
	Combined oxidative phosphorylation deficiency 3 (CoxPD3)		*TSFM*		X		
	Leucoencephalopathy with brain stem and spinal cord involvement and lactate elevation (LBSL)		*DARS2*	X			
	Coenzyme Q10 deficiency primary, 8		*COQ7*		X		
	Cataract, growth hormone deficiency, sensory neuropathy and hearing loss, and skeletal dysplasia (CAGSSS)		*IARS2*	X	X		
	Mitochondrial DNA depletion syndrome type 6 (MTDPS6), also known as Navajo neuropathy		*MPV17*	X	X	X	
	Mitochondrial DNA depletion syndrome type 3 (MTDPS3), also known as DGUOK deficiency		*DGUOK*				[1]
	Mitochondrial DNA depletion syndrome type 5 (MTDPS5), also known as Booth–Haworth–Dilling Syndrome		*SUCLA2*		X		
	Parkinsonism, deafness, and sensory-motor axonal neuropathy		*MT-RNR1*	X			
	Dominant optic atrophy plus (DOA+), also known as Behr syndrome		*OPA1*	X	X		
	Dominant optic atrophy (DOA), also known as Costeff syndrome		*OPA3*	X			
	Mitochondrial Complex 4 deficiency, Nuclear Type 11		*COX20*		X		
	Mitochondrial Complex 5 deficiency		*MTATP8*				[1]
	Congenital disorder of deglycosylation 1 (CDDG1)		*NGLY1*				[1]
	Ornithine aminotransferase deficiency		*MT-TK*				[3]
	Adult-Onset Chronic Progressive External Ophthalmoplegia with Mitochondrial Myopathy		*RNASEH1*		X		
DNA repair/replication	Ataxia-oculomotor apraxia 1 (OA1), also known as ataxia early-onset with oculomotor apraxia and hypoalbuminemia (EAOH)		*APTX*	X	X		
	Ataxia-oculomotor apraxia 2 (AOA2), also known as spinocerebellar ataxia autosomal recessive 1 (SCAR1), and spinocerebellar ataxia autosomal recessive with axonal neuropathy 2 (SCAN2)		*SETX*	X	X	X	
	Ataxia telangiectasia		*ATM*	X	X		
	Cockayne syndrome, also known as Neill–Dingwall Syndrome		*ERCC6, ERCC8*	X	X		
	Xeroderma Pigmentosum		*XPA*	X			
Aminoacidopathies	Homocysteine methylation disorders (cobalamin and methylenetetrahydrofolate reductase)		*MMACHC*	X			
			*MTHFR*				[2,3,14]
	Serine deficiency		*PGDH*				[3]
	Tyrosinaemia type 1		*FAH*	X			
Inflammatory	CD59 deficiency		*CD59*	X			
	Aicardi–Goutieres syndrome		*RNASEH2A, RNASEH2B, RNASEH2C, TREX1, ADAR1, IFH1, SAMHD1*				[1]
	Adenosine deaminase 2 deficiency (DADA2)		*ADA2*				[133]
Vitamin-related disorders	Brown–Vialetto–Van Laere (BVVL), also known as riboflavin transporter deficiency (RTD)		*SLC52A2, SLC52A3*	X	X		
	Biotinidase deficiency		*BTD*				[2,3]
	Abetalipoproteinemia		*MTTP*	X	X		
	Ataxia with isolated vitamin E deficiency (AVED)		*TTPA*	X	X		
	Cerebral folate deficiency (CFD)		*FOLR1*				[2]
	Thiamine metabolism dysfunction syndrome 4 (THMD4)		*SLC25A19*	X	X		
Amyloidosis	Familial amyloid polyneuropathy (FAP) type I-II		*TTR*	X	X	X	
	Familial amyloid polyneuropathy (FAP) type III, also known as Van Allen or Iowa type		*APOA1*		X		
	Familial amyloid polyneuropathy (FAP) type IV		*GSN*		X		
Giant axonal neuropathy	Giant axonal neuropathy 2		*DCAF8*			X	
	Giant axonal neuropathy 1		*GAN*	X	X	X	
Other	Tangier		*ABCA1*	X	X		
	Xanthomatosis cerebrotendinous (XCT)		*CYP27A1*	X	X		
	Charlevoix–Saguenay Spastic Ataxia (ARSACS)		*SACS*	X	X		
	Chorea acanthocytosis, also known as choreoacanthocytosis		*VPS13A*	X			
	McLeod syndrome		*XK*	X			
	Spinal and bulbar muscular atrophy, also known as Kennedy disease		*AR*				[1]
	X-fragile tremor and ataxia syndrome (FXTAS)		*FMR1*		X		
	Cerebellar ataxia with neuropathy and vestibular areflexia syndrome (CANVAS)		*RFC1*				[1,3,14]
	Posterior column ataxia and retinitis pigmentosa (PCARP)		*FLVCR1*	X	X	X	
	Polyneuropathy, hearing loss, ataxia, retinitis pigmentosa, and cataract (PHARC)		*ABHD12*	X			
	Pelizaeus–Merzbacher		*GJC2*	X			
			*PLP1*		X		
	Waardenburg syndrome		*SOX10*	X	X	X	
	Adult polyglucosan body disease (APBD), also known as glycogenosis type IV, glycogen storage disorder type 4, and Andersen disease		*GBE1*		X		
	Neurofibromatosis type II		*NF2*		X		
	Agenesis of the corpus callosum with peripheral neuropathy (ACCPN), also known as Andermann syndrome		*SLC12A6*	X	X	X	
	Triple A syndrome		*AAAS*		X		
	Congenital disorder of glycosylation type 1A (CDG1A)		*PMM2*	X			
	Congenital cataracts, facial dysmorphism, and neuropathy (CCFDN)		*CTDP1*	X	X	X	
	Leukodystrophy hypomyelination and congenital cataract (HLD5 HCC)		*FAM126A*	X	X		
	Myopathy congenital, deafness and neuropathy (CMND)		*SPTBN4*	X			
	Congenital insensitivity to pain		*CLTCL1*		X	X	
	Merosin-deficient congenital muscular dystrophy		*LAMA2*		X		
	Neurodegeneration with brain iron accumulation 2A (NBI2A), also known as infantile neuroaxonal dystrophy (INAD)		*PLA2G6*		X		
	Familial dysautonomia, also known as hereditary sensory autonomic neuropathy with intellectual disability (HSAN9) and hereditary spastic paraplegia 49		*TECPR2*				[1,14]
	Hypomyelinating leukodystrophy 6, also known as TUBB4A-related leukodystrophy		*TUBB4A*				[1]
	Pontocerebellar hypoplasia type 1B (PCH1B)		*EXOSC3*				[1]
	Pontocerebellar hypoplasia (PCH9)		*AMPD2*				[1]
	Action myoclonus–renal failure syndrome (AMRF)		*SCARB2*				[1,14]
	Kyphoscoliotic type of Ehlers–Danlos syndrome (EDS 6)		*PLOD1*				[1]
	Familial visceral amyloidosis		*B2M*				[1]

## Appendix B

**Table A2:** Information on inherited neuropathies for which therapies have been tested clinically over the last five years.

Diseases for Which Trials Were Found	Gene	Inheritance	Physiopathology/Comments	Clinical Aspects	Type of Neuropathy	Standard of Care
Hereditary transthyretin amyloidosis- polyneuropathy (ATTRv-PN)	*TTR*	AD	Mutation changes the tetrameric structure of the TTR protein, leads to dissociation into misfolded monomer subunits, which then accumulate as amyloid deposits in different tissues including peripheral nerves [134].	Systemic disease including cardiomyopathy, renal, and ocular involvement [134].	Sensorimotor predominantly axonal, length-dependent and autonomic neuropathies [134]	- Liver transplantation was standard of care until recently: effectiveness of the transplanted organ is impaired by the continuous deposition of wild-type transthyretin amyloid [135]. - Alternative therapeutic approaches include transthyretin tetramer stabilizers (tafamidis) and *TTR* gene silencers (inotersen and patisiran) [96,97].
Spinocerebellar ataxia type 38 (SCA 38)	*ELOVL5*	AD	More than 40 distinct subtypes have been identified: mechanism depends on the variant [136].	Starts at around age 40 with cerebellar symptoms. As the disease progresses, patients may present with hyposmia, hearing loss and pes cavus without paresia [114].	Predominantly sensorimotor axonal polyneuropathy [114]	- Symptomatic.
Spinocerebellar ataxia type 2 (SCA2)	CAG trinucleotide repeat expansion in the *ATXN2* gene	AD	More than 40 distinct subtypes have been identified: mechanism depends on the variant [136].	Slow saccades, ataxia, tremor, parkinsonism [1].	Motor-predominant axonal neuropathy [1]	- Symptomatic.
Familial dysautonomia	*ELP-1*	AR	Defect in baroreceptor neurons in cranial nerves IX and X [137].	Variability in blood pressure among other dysautonomia symptoms [137].	Small-fibre neuropathy [137]	- Symptomatic: anxiolytics (diazepam), clonidine, standard antihypertensives [41].
Hereditary sensory and autonomic neuropathy type 1 (HSAN1)	*SPTLC1* and *SPTLC2*	AD	Mutation reduces the affinity of the serine palmitoyltransferase enzyme for its normal substrate, serine and increases affinity for alanine and glycine, leading to the production of abnormal neurotoxic 1-deoxysphingolipids [138].	Dysautonomia symptoms, skin ulcers, muscle weakness, sensory loss, and neuropathic pain [139].	Small-fibre and sensory axonal neuropathy [139]	- Symptomatic.
Ataxia-Telangiectasia (A-T)	*ATM*	AR	Mutation of *ATM* gene impairs DNA repair [140].	Ataxia and oculomotor apraxia beginning in early infancy, dystonia and/or chorea, conjunctival telangiectasia, susceptibility to infections and malignancies [1].	Sensory axonal neuropathy [1,141]	- Symptomatic.
Charcot–Marie–Tooth 1A (CMT1A)	*PMP22*	AD	Duplication of the *PMP22* gene, leading to over-expression of PMP22 protein, and gain-of-function phenotype [142,143].	Neuropathy is the sole or predominant clinical feature of the disease [4].	Demyelinating sensorimotor neuropathy [4]	- Symptomatic.
Metachromatic leukodystrophy (MLD)	*ASA*	AR	Deficient activity of the lysosomal enzyme arylsulfatase A, results in accumulation of sulfatide in cells of both peripheral and central nervous systems [144].	Three forms are commonly described depending on age of onset: late-infantile, around 2 years; juvenile, between 3 and 16 years; and adult, after 16 years. The younger onsets are the most severe forms, with clinical regression. Adults present with optic atrophia, cognitive impairment, ataxia, and paresia [144].	Demyelinating neuropathy [1]	- Allogeneic haematopoietic stem-cell transplantation has been reported to slow disease progression in some patients, especially in late-onset presymptomatic phenotype [145,146].
X-linked adrenoleukodystrophy (X-ALD)	*ABCD1*	X-linked	Mutation in protein involved in the transmembrane transport of very-long-chain fatty acids leads to accumulation of these fatty acids in different tissues [147].	Very broad spectrum. Symptoms range from adrenocortical insufficiency only to cerebral adrenoleukodystrophy to adrenomyeloneuropathy (AMN). Most adult patients present with AMN, which involves myelopathy, neuropathy, and possible cerebral involvement [147].	Sensorimotor axonal (sometimes demyelinating) neuropathy [1]	- Symptomatic.
Spinal and bulbar muscular atrophy (SBMA), also known as Kennedy disease	Expansion of CAG repeat in the *AR* gene	X-linked	Mutation of the receptor for androgen results in degeneration of lower motor neurons and skeletal muscles [148].	Muscle weakness, atrophy, fasciculations, occasionally androgen insensitivity [1,148].	Motor neuropathy [1]	- Symptomatic.
Adult polyglucosan body disease (APBD)	*GBE1*	AR	Mutation of *GBE1* results in glycogen branching enzyme deficiency, which causes polyglucosan body accumulation in various tissues including peripheral nerves and cerebral white matter [149].	Cognitive impairment, spasticity, bladder dysfunction [149].	Sensorimotor axonal-predominant neuropathy [1,3].	- Symptomatic.
Fabry disease (FD)	*GLA*	X-linked	Mutation of *GLA* results in α-galactosidase A activity deficiency, leading to accumulation of globotriaosylceramide (Gb3) and its deacylated form globotriaosylsphingosine (lyso-Gb3) in plasma and different cell types. The severity of phenotype depends on the level of galactosidase A activity [150].	Multisystemic disease including cardiomyopathy, renal failure, and angiokeratoma [150].	Sensory axonal and small-fibre neuropathy [1,3]	- ERT with recombinant human α-Gal A. - Chaperone therapy (migalastat) for amenable mutations [150,151].
Acid sphingomyelinase deficiency (ASMD)	*SMPD1*	AR	Mutation results in lysosomal enzyme acid sphingomyelinase activity deficiency, leading to sphingomyelin accumulation in various tissues, including the macrophage–monocyte system [152].	Phenotypic spectrum ranges from severe infantile presentation with early death to subacute/chronic neurovisceral forms. Patients present with hepatosplenomegaly, thrombocytopenia, and interstitial lung disease [152].	Mostly demyelinating polyneuropathy [127,128]	- Symptomatic.
Mitochondrial encephalomyopathy, lactic acidosis, and stroke-like episodes (MELAS)	*MT-TL1*	Maternal inheritance	Mutation results in mitochondrial dysfunction [153].	Stroke-like episodes [153].	Subclinical polyneuropathy, predominantly sensory axonal [1,154]	- Symptomatic (anticonvulsivant drugs).
Gaucher disease type 1 (GD1)	*GBA1*	AR	Mutation results in *β*-glucocerebrosidase enzyme activity deficiency, leading to lysosomal glucocerebroside accumulation in various tissues [155].	Hepatosplenomegaly, thrombocytopenia, anaemia, bone disease, growth failure [155]	Sensorimotor axonal neuropathy [14,118]	- ERTs with recombinant *β*-glucocerebrosidase are approved in several countries for paediatric and adult patients; these include imiglucerase (Cerezyme, Genzyme Corporation), velaglucerase alfa (VPRIV, Shire Human Genetic Therapies, Inc.), and taliglucerase alfa (Elelyso, Pfizer; Protalix, BioTherapeutics). - The SRT miglustat (Zavesca, Janssen Pharmaceutica NV) is approved for adult GD1 patients for whom ERT is considered unsuitable. - The SRT eliglustat (Cerdelga, Sanofi Genzyme) is approved for adults with GD1 who are CYP2D6 extensive, intermediate, or poor metabolizers [120].
Acute intermittent porphyria (AIP)	*HMBS*	AD	Porphobilinogen deaminase enzyme activity deficiency results from *HMBS* mutation, leading to depletion of free heme. This results in up-regulation of the delta-aminolevulinic acid synthase and overproduction of toxic heme intermediates that are thought to cause disease manifestations [156].	Acute neurovisceral attacks characterized by abdominal pain and mental status changes (seizures, psychosis) [157].	Autonomic neuropathy and acute motor axonal neuropathy resembling Guillain–Barré syndrome during attacks. Subjects not treated promptly or with recurrent attacks may develop chronic neuropathies, typically motor axonal polyneuropathies [1,158].	- Management of attacks relies on hemin and glucose use as well as avoidance of precipitating factors [159]. - Givosiran (GIVLAARI, Alnylam Pharmaceuticals, Inc.) is approved in patients ≥ 12 years with ≥4 attacks within 12 months [111].
Friedreich ataxia (FRDA)	Trinucleotide (GAA) repeat expansions in the *FXN* gene	AR	Reduction in the amount of functional mitochondrial frataxin protein results from repeat expansion in *FXN*, leading to mitochondrial dysfunction and increased sensitivity to oxidative stress [160].	Ataxia, cardiomyopathy, diabetes, and loss of visual and sensorineural hearing function [161].	Sensory axonal neuropathy [1,3]	- Omaveloxolone was recently FDA-approved (brand name Skyclarys, Reata Pharmaceuticals).
Bile acid synthesis disorder (BASD)	Several causative genes	Depends on condition	Deficiency or lack of activity in enzymes that catalyse the conversion of cholesterol to bile acids (i.e., single enzyme defects) or defects in oxidation and shortening of the cholesterol side chain caused by generalized peroxisomal dysfunction (Zellweger spectrum disorder) [162].	Liver dysfunction [162]	Neuropathies are seen in single-enzyme defects such as in patients with cerebrotendinous xanthomatosis (predominantly sensory axonal neuropathy), with alpha-methylacyl-CoA racemase deficiency (axonal or demyelinating neuropathy) [1] and in subjects with Zellweger spectrum disorder who survive into adulthood (mostly demyelinating neuropathies) [163].	- Bile acid supplementation; cholic acid or chenodeoxycholic acid depending on the subtype of the disease. - Cholic acid capsules (CHOLBAM, Travere Therapeutics) were FDA approved in 2015 [86].
Fragile-X associated tremor/ataxia syndrome (FXTAS)	Premutation expansion (59-199 CGG) in the *FMR1* gene	X-linked	Expansion results in overproduction of *FMR1* mRNA [164]	Late-onset intention tremor, ataxia, parkinsonism, cognitive decline [164]	Sensory axonal neuropathy [1,3,164]	- Symptomatic.

Abbreviations: TTR, transthyretin; AD, autosomal dominant; AR, autosomal recessive; ERT, enzyme replacement therapy; SRT, substrate reduction therapy; FDA, US Food and Drug Administration.

## Appendix C

**Table A3:** Risk of bias.

Reference of Study	JADAD Score	Reference of Study	JADAD Score	Reference of Study	JADAD Score	Reference of Study	JADAD Score
[18]	1	[42]	5	[65]	5	[85]	1
[19]	1	[43]	5	[66]	5	[87]	1
[20]	1	[44]	1	[67]	1	[88]	2
[22]	5	[45]	1	[68]	1	[89]	5
[23]	5	[46]	1	[69]	1	[90]	5
[24]	3	[48]	1	[70]	1	[91]	1
[25]	1	[49]	4	[71]	1	[92]	1
[26]	3	[50]	5	[72]	0	[93]	1
[27]	1	[51]	1	[73]	1		
[29]	1	[53]	2	[74]	5		
[31]	4	[54]	1	[75]	1		
[32]	4	[55]	0	[76]	0		
[33]	4	[57]	0	[77]	4		
[34]	4	[58]	1	[78]	5		
[35]	1	[59]	1	[79]	5		
[36]	1	[60]	1	[80]	1		
[37]	1	[61]	1	[81]	5		
[38]	1	[62]	4	[82]	5		
[40]	1	[63]	1	[83]	5		
[41]	5	[64]	0	[84]	5		
